# Functionalized Folate-Modified Graphene Oxide/PEI siRNA Nanocomplexes for Targeted Ovarian Cancer Gene Therapy

**DOI:** 10.1186/s11671-020-3281-7

**Published:** 2020-03-06

**Authors:** Yunfei Wang, Guoping Sun, Yingying Gong, Yuying Zhang, Xiaofei Liang, Linqing Yang

**Affiliations:** 1 Department of Gynecology, Affiliated Hospital of Jining Medical University, Jining Medical University, 89 Guhuai Road, Jining, 272029 Shandong People’s Republic of China; 2 Department of Pharmacy, Qingdao Seventh People’s Hospital, 299 Nanjing Road, Qingdao, 266034 Shandong People’s Republic of China; 3 Department of State Key Laboratory of Oncogenes and Related Genes, Shanghai Cancer Institute, Renji Hospital, Shanghai Jiaotong University School of Medicine, Shanghai, 200032 People’s Republic of China

**Keywords:** Ovarian cancer, Folate, GO, Target, Gene therapy, Lysosomal escape

## Abstract

Gene therapy is emerging as a valid method for the treatment of ovarian cancer, including small interfering RNA (siRNA). Although it is so powerful, few targeting efficient gene delivery systems seriously hindered the development of gene therapy. In this study, we synthesized a novel gene vector PEG-GO-PEI-FA by functionalized graphene oxide (GO), in which folic acid (FA) can specifically bind to the folate receptor (FR), which is overexpressed in ovarian cancer. Characterizations of the nanocomplexes were evaluated by dynamic light scattering (DLS), atomic force microscopy (AFM), and Fourier transform infrared spectroscopy (FTIR). The siRNA condensation ability and stability were assessed by agarose gel electrophoresis. Cellular uptake efficiency and lysosomal escape ability in ovarian cancer cells were investigated by confocal laser scanning microscopy. Furthermore, cellular biosafety of the system and inhibitory of the siRNA tolerability were evaluated by CCK-8 assay. The size of the PEG-GO-PEI-FA nanocomplexes was 216.1 ± 2.457 nm, exhibiting mild cytotoxicity in ovarian cancer cells. With high uptake efficiency, PEG-GO-PEI-FA can escape from the lysosome rapidly and release the gene. Moreover, PEG-GO-PEI-FA/siRNA can effectively inhibit the growth of ovarian cancer cells. By and large, the PEG-GO-PEI-FA/siRNA may offer a promising strategy for siRNA delivery in the treatment of FR-positive ovarian carcinoma or similar tumors.

## Introduction

Ovarian cancer is the leading gynecological cause of death in the world and is usually associated with poor clinical outcomes due to the difficulties of early diagnosis and therapy [1–3]. Unfortunately, conventional chemotherapeutic agents exhibit inherent limitations such as nonspecific distribution, poor bioavailability, rapid blood clearance, and poor solubility in physiological environments [4]. Over the last several decades, gene therapy has been investigated as a potential approach for the treatment of various genetic disorders, including cancers [5–8]. However, the lack of a safe, highly efficient, and selective gene delivery carrier has hindered its advancement to clinical utility. Currently, there are two categories of gene vectors: viral and non-viral vectors. Although viral vectors have shown high transfection efficiency, several drawbacks limit their clinical application, for example, severe immune inflammatory reactions and the risk of recombination with wild-type viruses [9, 10]. In contrast, non-viral vectors have an extensive delivery capacity, low immunogenicity, and structural flexibility, making them excellent alternatives to viral vectors [11–13]. With the rapid development of nanotechnology, many nanoparticles have been explored for potential use as gene delivery carriers [14, 15]. Here, this work had designed a novel non-viral vector PEG-GO-PEI-FA that can be delivered efficiently to tumor tissues.

Graphene oxide (GO) is the derivative of graphite that be produced by the multi-step oxidation, ultrasonic, and purification of graphite [16]. The GO surface contains epoxy, hydroxyl, and carboxyl groups [17]. The oxygen functional groups on its surface give GO excellent biocompatibility that allows it to form a stable suspension in the water and organic solvents, facilitating chemical modifications and functionalization [18, 19]. So, GO has been widely used for photothermal cancer therapy, drug and gene delivery, and biosensors [20–22]. Polyethylene glycol (PEG) is a safe, non-toxic, biodegradable material; it has been approved by the US Food and Drug Administration as a hydrophilic drug carrier [23]. The GO functionalization with PEG can improve the stability under physiological conditions, extend the cycle time of GO nanomaterial in the body, and improve GO nanomaterial pharmacokinetics for better tumor targeting [24, 25]. Polyethyleneimine (PEI) has become a gold standard of non-viral vectors because of its high transfection efficiency in various cell lines [26]. However, PEI shows severe cytotoxicity and poor biocompatibility, thereby limiting its clinical applications. In this study, we observed a significant decrease in cytotoxicity when PEI was grafted to the surface of GO. Folate receptor is over-expressing on various cancer cells surface, especially in ovarian cancer cells both before and after chemotherapy [27]. For targeted gene delivery, folic acid molecules were covalently bonded with GO to target folate receptors.

Overall, the main aim of the present study was to explore a new targeting GO-based gene delivery system to increase the utility of siRNA-based gene therapy for ovarian cancer. The obtained positively charged PEG-GO-PEI-FA could be loaded with siRNA for gene delivery. Its successful synthesis, characterization, effective cellular internalization, and in vitro anticancer effect were analyzed using various techniques. Besides, the safety of this system was also evaluated in vitro.

## Materials and Methods

### Materials

Graphene oxide was obtained from Suzhou Carbon Fung Graphene Technology Co. Ltd. (Suzhou, China). Folic acid (FA), *N*-hydroxysuccinimide (NHS), and fluorescein isothiocyanate (FITC) were purchased from Sigma Aldrich Trading Co. Ltd. (Shanghai, China). Branched PEI 25K, PEG (MW = 2000), agarose, and 1-(3-dimethylaminopropy l)-3-ethyl carbodiimide hydrochloride (EDC·HCL) were purchased from Adama Reagent Co. Ltd. (Shanghai, China). Phosphate-buffered solution (PBS), Dulbecco’s phosphate-buffered saline (DPBS), and all other chemicals were purchased from Sinopharm Chemical Reagent Co. Ltd. (Shanghai, China). All other chemicals were of reagent grade. RPMI-1640 medium (Gibco), fetal bovine serum (FBS) (Gibco), trypsin (Gibco), and 20× TAE buffer were purchased from Thermo Fisher Scientific Co. Ltd. (China). Cell counting kit8 (CCK-8) was purchased from Shanghai Yisheng Biological Co. Ltd. (Shanghai, China). LysoTrackerRed, 4% paraformaldehyde, 4′-6-diamidino-2-phenylindole (DAPI), and Lipofectamine 2000 transfection reagents were purchased from Invitrogen Co. Ltd. (China). Dil fluorescence probe (Cat number: C1036) was purchased from Beyotime Co. Ltd. Anti-PLK1 (PLK1-homo-581) short interfering RNA (siRNA) was synthesized by Shanghai GenePharma Co. Ltd. (Shanghai, China).

### Preparation of PEG-GO-PEI-FA

The synthesis of PEG-GO-PEI-FA is according to the strategy shown in Figure S2 (A). Firstly, 10 mL GO aqueous suspension (1000 μg/mL) was continuously bath sonicated at 50 W for 2 h. Then, 1 mL EDC·HCL (5000 μg/mL) and NHC (5000 μg/mL) were added and intermittently sonicated for 30 min at 100 W, followed by adding PEG (10 mg) sonicated for 30 min and stirred on a magnetic stirrer for 6 h at 0 °C. To remove the unreacted reagents and get GO-PEG solution, the mixture was dialyzed for 24 h in distilled water with a dialysis membrane (MWCO, 3500 Da). Then, GO-PEG solution was sonicated for 30 min, 1 mL EDC·HCL (5000 μg/mL) and NHC (5000 μg/mL) were added and sonicated for 30 min again, followed by adding PEI 25K (5000 μg/mL) 2 mL. The mixture was sonicated for 30 min and stirred for 6 h at 0 °C. Subsequently, to get the PEG-GO-PEI, the mixture was dialyzed for 24 h in the distilled water with a dialysis membrane (MWCO, 3500 Da) again. The PEG-GO-PEI solution was sonicated for 60 min, 1 mL EDC·HCL (5000 μg/mL) and NHC (5000 μg/mL) was added and sonicated for 30 min again, followed by adding 10 mg FA to the solution. The solution was sonicated for 30 min and stirred for 12 h at 0 °C and dialyzed for 48 h to get PEG-GO-PEI-FA according to the abovementioned procedure. The solution of nanocomplexes was dried by vacuum freeze dryer (Biosafer-10D).

### Characterization

The surface zeta potential and average particle size of the nanocomplexes at water solution were measured by dynamic light scattering (DLS, Malvern Zetasizer Nano ZS, UK). The morphologies of the nanocomplexes were observed using atomic force microscope in the atmosphere (AFM, MuLtimode Nanoscope IIIa, Germany). Fourier transform infrared spectroscopy (FTIR) spectra were acquired by Thermo Nicolet 6700 FTIR spectrograph, using the KBr pellet at the range of 400–4000 cm^−1^. The UV-Vis absorption spectra of the nanocomplexes were measured by UV-Vis spectrophotometer (UV, EV300, USA). The Raman of the nanocomplexes were measured by dispersive Raman microscope (Senterra R200-L, Germany) with an excitation wavelength at 532 nm.

### In Vitro Biosecurity Assay

The SKOV3 (human ovarian cancer cells) was purchased from Keygen (China). Cells were maintained in RPMI-1640 medium, supplemented with 10% FBS, 100 U/mL penicillin, 100 g/mL streptomycin, and cultured at 37 °C under a humidified atmosphere containing 5% CO_2_. In this study, the cytotoxicity of materials was analyzed in SKOV3 cells by CCK-8 assay. Briefly, the cells were seeded in 96-well plate at the density of 6 × 10^3^ cells/well in 100 μL 1640 medium containing 10% FBS and then incubated at 37 °C for 12 h in a humidified atmosphere containing 5% CO_2_. Then, GO, GO-PEG, PEG-GO-PEI, and PEG-GO-PEI-FA were added at final concentrations ranging from 10 to 1000 μg/mL and incubated with the cells for additional 12 h. Another, GO, GO-PEG, PEG-GO-PEI, and PEG-GO-PEI-FA were incubated with different time from 4 to 24 h at the concentrations 100 μg/mL. Subsequently, 10 μL of CCK-8 was added to each well and incubated for another 3 h at 37 °C. After that, the absorbance at 450 nm was measured on a microplate reader. Each group experiment was repeated three times. The relative cell viability (%) related to control cells cultured in media was calculated as (absorbance of sample − absorbance of blank)/(absorbance of control − absorbance of blank) × 100%.

### Agarose Gel Retardation Assay

The 20× TAE buffer solution was diluted with DEPC-treated water to make 1% agarose solution. Then, the solution was heated for 5 min by a microwave oven to completely dissolve the agarose. When the temperature of the agarose solution dropped down to 55 °C, ethidium bromide (EB, 0.5 μg/mL) solution in the volume ratio of 10,000:1 was added and mixed uniformly, subsequently, the agarose solution by cooling to form agarose gel. The PEG-GO-PEI, PEG-GO-PEI-FA, and siRNA mixture at different weight ratios were added to the hole. The gel was run at 110 V and 40 mA for about 60 min, and then observed and analyzed by ultraviolet imager.

### Cell Uptake Studies of PEG-GO-PEI-FA

To investigate the cell uptake, the nanocomplexes were labeled with FITC according to previously described method with a little modification [28]. Briefly, the solution of nanocomplexes (approximately 1 mg/mL, 2.0 mL) was mixed with 0.2 mL FITC (26 mM) dissolved in DMSO and then stirred over night at room temperature. The resulting mixtures were dialyzed through 5000 MWCO membrane to remove unlabeled FITC and then lyophilized. The cells were seeded in a 6-well plate (containing the coverslips) at the density of 2 × 10^5^ cells/well in 2 mL 1640 medium containing 10% FBS and then incubated at 37 °C for 12 h in a cell culture incubator. Then, the medium in each well was replaced with serum-free 1640 medium. Subsequently, the cells were treated with different nanocomplexes/FITC. Cells treated without the nanocomplexes are the control group, and cells treated with PEI 25 K are the parallel control group. After incubation for 4 h at 37 °C in a humidified atmosphere containing 5% CO_2_, the liquid of the culture plate was removed, the cells were washed twice with cold DPBS (pH = 7.4), then 20 μL DAPI and 5 μL Dil (cytomembrane dye) were added to each well and incubated 20 min at 37 °C under the dark. Subsequently, the DAPI and Dil of the culture medium were removed and washed twice with cold DPBS (pH = 7.4), and the coverslips of cells were taken out and fixed on a glass slide with Permount TM Mounting Medium. Whereafter, the fluorescence signal of the slides of cells was observed by Confocal Laser Scanning Microscope (CLSM) and analyzed the cell uptake of nanocomplexes.

### Endosomal Escape and Penetration into Nucleus

To visualize the cellular distribution, endosomal escape, and the penetration of nanocomplexes, SKOV3 cells were seeded in 12-well plate (containing the coverslips) at the density of 1 × 10^5^ cells/well in 2 mL 1640 medium containing 10% FBS and then incubated for 12 h (37 °C, 5% CO_2_). Then, the medium in each well was replaced with 1 mL/well serum-free 1640 medium. Subsequently, the different nanocomplexes (100 μg/mL) labeled by FITC were added in 12-well plate for 1 mL/well. After 6 h, the liquid was removed, cells were washed twice with cold DPBS (pH = 7.4), and the 12-well plate was added LysoTracker Red (5 μg/mL) for 50 μL/well. After incubation for 15 min at 37 °C in a humidified atmosphere containing 5% CO_2_, the liquid of the 12-well plate was removed, the cells were washed twice with cold DPBS (pH = 7.4), then 20 μL of DAPI was added to each well and incubated for 20 min at 4 °C under the dark. Next, the DAPI liquid was removed and washed twice with cold DPBS (PH = 7.4). After, with 4% paraformaldehyde fixed 15 min at room temperature, and the coverslips were taken out and fixed on a glass slide with Permount TM Mounting Medium. The fluorescence signal of the slides was observed by confocal laser scanning microscopy and analyzed the lysosomal escape and penetration into the nucleus of nanocomplexes [29].

### Cyto-inhibition Analysis

We analyzed the cell inhibition effect of PEG-GO-PEI/siRNA and PEG-GO-PEI-FA/siRNA using the CCK-8 assay. Shortly, the cells were seeded in 96-well plate at a density of 6 × 10^3^ cells/well in 100 μL 1640 medium containing 10% FBS and then incubated at 37 °C for 12 h in a humidified atmosphere containing 5% CO_2_. Then, PEG-GO-PEI and PEG-GO-PEI-FA were added at final concentrations ranging from 10 to 500 μg/mL and incubated with the cells for an additional 12 and 24 h. Then, PEG-GO-PEI/siRNA and PEG-GO-PEI-FA/siRNA were incubated with cells from 4 to 48 h at a concentration of 100 μg/mL. The siRNA was transfected with Lipofectamine 2000 and untreated as the control group. Subsequently, 10 μL of CCK-8 was added to each well and incubated for another 3 h at 37 °C. The absorbance was measured at 450 nm. Each group experiment was repeated three times. The inhibitory rate (%) was calculated as 1 − (absorbance of sample − absorbance of blank)/(absorbance of control − absorbance of blank) × 100%.

### Statistics

Each experiment testing was repeated three times, and the data were analyzed using SPSS 17.0 software. One-way ANOVA test for multiple-group analysis and unpaired Student’s *t* test for two-group analysis were used for comparison. The data were expressed as mean and standard deviation (SD), and the statistical significance was set at *p* < 0.05.

## Results and Discussion

### Synthesis of PEG-GO-PEI-FA

The expression of FR was firstly analyzed in different cancer cell lines based on the Cancer Cell Line Encyclopedia (CCLE; http://portals.broadinstitute. org/ccle) database [30] and different tissues of ovary based on the Gene Expression Profiling Interactive Analysis (GEPIA2; http://gepia2.cancer-pku.cn) database [31]. The results were consistent with previous research (Figure S1). Therefore, we chose FA as a targeting ligand for gene delivery.

**Fig. 1:**
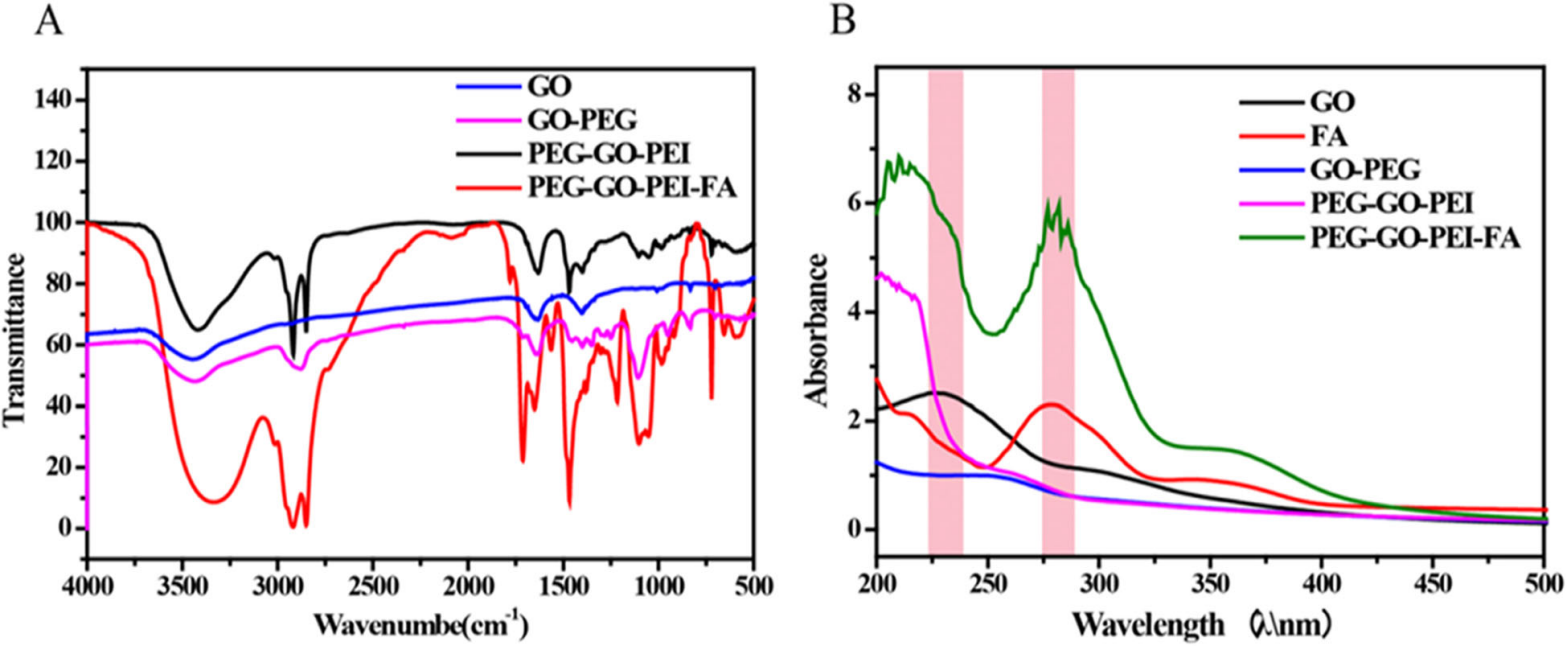
The successful synthetic analysis of PEG-GO-PEI-FA using Fourier transform infrared spectroscopy (FTIR) and UV-Vis spectrophotometer. **a** The FTIR spectra of GO, GO-PEG, PEG-GO-PEI, and PEG-GO-PEI-FA. **b** The UV-Vis absorption spectra of GO, FA, GO-PEG, PEG-GO-PEI, and PEG-GO-PEI-FA

Graphene oxide certainly bears many oxygen-containing groups including carboxyls at the edge, hydroxyls, and epoxy groups on the basal plane produced by the oxidation process [32]. To obtain nanoscale GO (NGO), GO was continuously cracked by bath sonication at 50 W for 2 h and then followed by covalent linking of PEG/PEI/FA to GO using EDC/NHS chemistry, as illustrated in Figure S2(A). Figure S2(B) showed the treatment route of the PEG-GO-PEI-FA/siRNA. The analysis of the chemical conjugation was carried out by FTIR differential spectra technology to obtain the spectral absorption peaks [33]. As shown in Fig. 1a, the existence of OH (3425 cm^−1^), C=O (1719 cm^−1^), and C=H (1350 cm^−1^) functional groups were found in GO and indicated the existence of hydroxyl and carboxyl in the surface of GO. CH (2885 cm^−1^) stretching vibration band could be found when the PEG reacted with GO via stable covalent bonds; this indicated PEG had been grafted to GO, and the PEG conjugation efficiency was about 6%. After PEI and FA reacted with GO, the NH (1590 cm^−1^), C–N (1420 cm^−1^) stretching vibration band was observed, indicating PEI and FA had been grafted to GO by esterification. These results demonstrated that the conjugation PEG-GO-PEI-FA had been successfully synthesized.

Figure 1b showed the UV-Vis absorption spectra of GO, FA, GO-PEG, PEG-GO-PEI, and PEG-GO-PEI-FA. The GO had an absorption peak of 223 nm. The FA had an absorption peak of 275 nm. The GO-PEG showed a smooth curve, indicating that PEG had conjugated with GO and made the mountain of GO more smooth. The PEG-GO-PEI had an absorption peak in 219 nm, illustrating PEI had grafted to the surface of GO-PEG. The PEG-GO-PEI-FA had an absorption peak in 219 nm and 275 nm, revealing FA had grafted to the PEG-GO-PEI. All of these results further demonstrated the successful synthesis of PEG-GO-PEI-FA.

### Characterization of PEG-GO-PEI-FA

The data given in Table 1 showed the particle size and zeta potential of the nanocomplexes. The particle size of nanocomplexes gradually increased to 218.4 nm while PEG, PEI, and FA grafted progressively to the surface of GO. The zeta potential changed from − 16.5 to + 17.5 mv when PEI connected to GO, which facilitated the ability to adsorb negatively charged DNA or RNA via electrostatic interaction and cellular uptake. The zeta potentials of PEG-GO-PEI-FA and PEG-GO-PEI-FA/siRNA were + 14.7 mv and + 14.5 mv, respectively. The zeta potentials revealed that PEG-GO-PEI-FA or PEG-GO-PEI-FA/siRNA were smaller than PEG-GO-PEI because of the negative charge of FA and siRNA. These indicated that PEG-GO-PEI-FA/siRNA could be adsorbed to the surface of cells by the charge interaction and be used by receptor-mediated endocytosis on the cytomembrane [34].

**Table 1:** The particle size and zeta potential of the functional NGO

Nanocarriers	Size (nm)	PDI	Zeta (mV)
GO	192.1 ± 2.135	0.191	− 22.7 ± 2.213
GO-PEG	200.2 ± 3.301	0.270	− 16.5 ± 3.134
PEG-GO-PEI	214.3 ± 2.013	0.172	17.5 ± 1.182
PEG-GO-PEI-FA	216.1 ± 2.457	0.284	14.7 ± 1.108
PEG-GO-PEI-FA/siRNA	218.4 ± 2.012	0.340	14.5 ± 1.216

Data were represented as mean ± SD

*PDI* polydispersity index

**Fig. 2:**
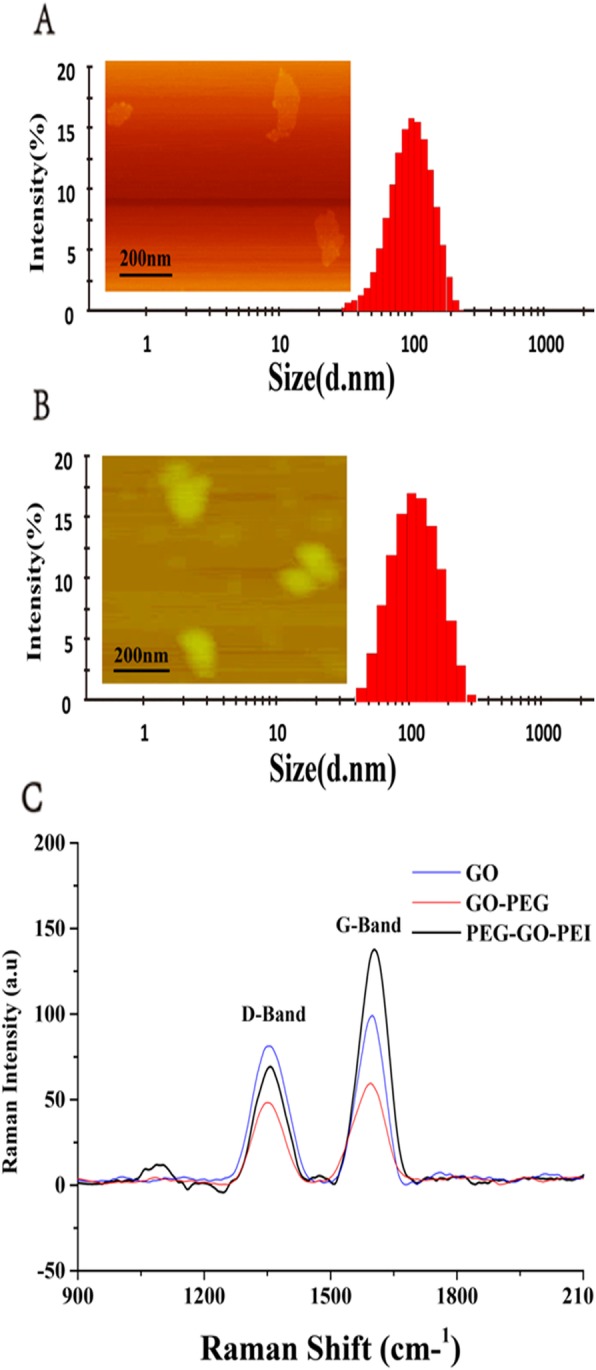
The characterization of the nanoscale delivery system. Atomic force microscopy (AFM) and dynamic light scattering (DLS) were used for characterizing morphology and size distributions: **a** GO and **b** PEG-GO-PEI-FA. Raman spectra for the analysis of the generated graphene oxide surface of GO, GO-PEG, and PEG-GO-PEI **c**

The surface morphology and particle size of the nanocarrier were also measured by AFM and DLS. As shown in Fig. 2a, the GO showed a smooth surface and sheet structure with a particle size of 192.1 nm. After PEG, PEI, and FA grafting to the surface of GO, the particle size of PEG-GO-PEI-FA increased to 216.1 nm, and many protuberances were observed on the surface of PEG-GO-PEI-FA (Fig. 2b), suggesting that a large number of decorations were immobilized onto the GO sheets. At the same time, the height of the PEG-GO-PEI-FA was more than GO, which is mainly due to the attachment of PEG, PEI, and FA on both planes of GO sheet.

Raman spectroscopy is one of the most commonly used to measure the characterization and structural properties of nanocarbon materials [35]. As shown in Fig. 2c, the Raman spectrum of GO showed the vibration band at 1600 cm^−1^ (G band) and 1354 cm^−1^ (D band), and the area ratio of ID/IG was 1.0385, indicating that the part SP^2^ hybridization of GO had been broken and formed hydroxyl and carboxyl. GO-PEG and PEG-GO-PEI showed the vibration band at 1595 cm^−1^ (G band) and 1352 cm^−1^ (D band), and the area ratio of ID/IG were 0.7737 and 0.5238. The area ratio of ID/IG gradually reduced following the PEG and PEI reaction with the GO; this indicated the PEG and PEI had grafted to the surface of the GO.

### In Vitro Biosecurity Analysis of the Different Functional NGO

The biosecurity issue of non-viral gene vectors has remained a significant challenge to clinical applications. The high positive charges not only brought about an excellent capacity to condense and protect genes but also led to severe cytotoxicity [36, 37]. To test the cytotoxicity of free siRNA nanocomplexes, CCK-8 assay was conducted with SKOV3 cells that had been incubated for 4, 8, 12, and 24 h with free nanocomplexes at different concentrations (from 10 to 1000 μg/mL). As shown in Figure S3, the nanocomplexes still had high cell viability in ovarian cancer at 1000 μg/mL and 24 h. The cytotoxicity of all nanocomplexes displayed a time- and concentration-dependent manner (viability of SKOV3 cells: greater than or equal to 84.38% for all nanocomplexes in 1000 μg/mL, and greater than or equal to 94.21% for all nanocomplexes at 24 h with SKOV3 cells). These results suggested that the nanocomplexes showed negligible cytotoxicity and could serve as a biocompatible gene vector.

### The Analysis of Nanocomplexes Combined with siRNA

**Fig. 3:**
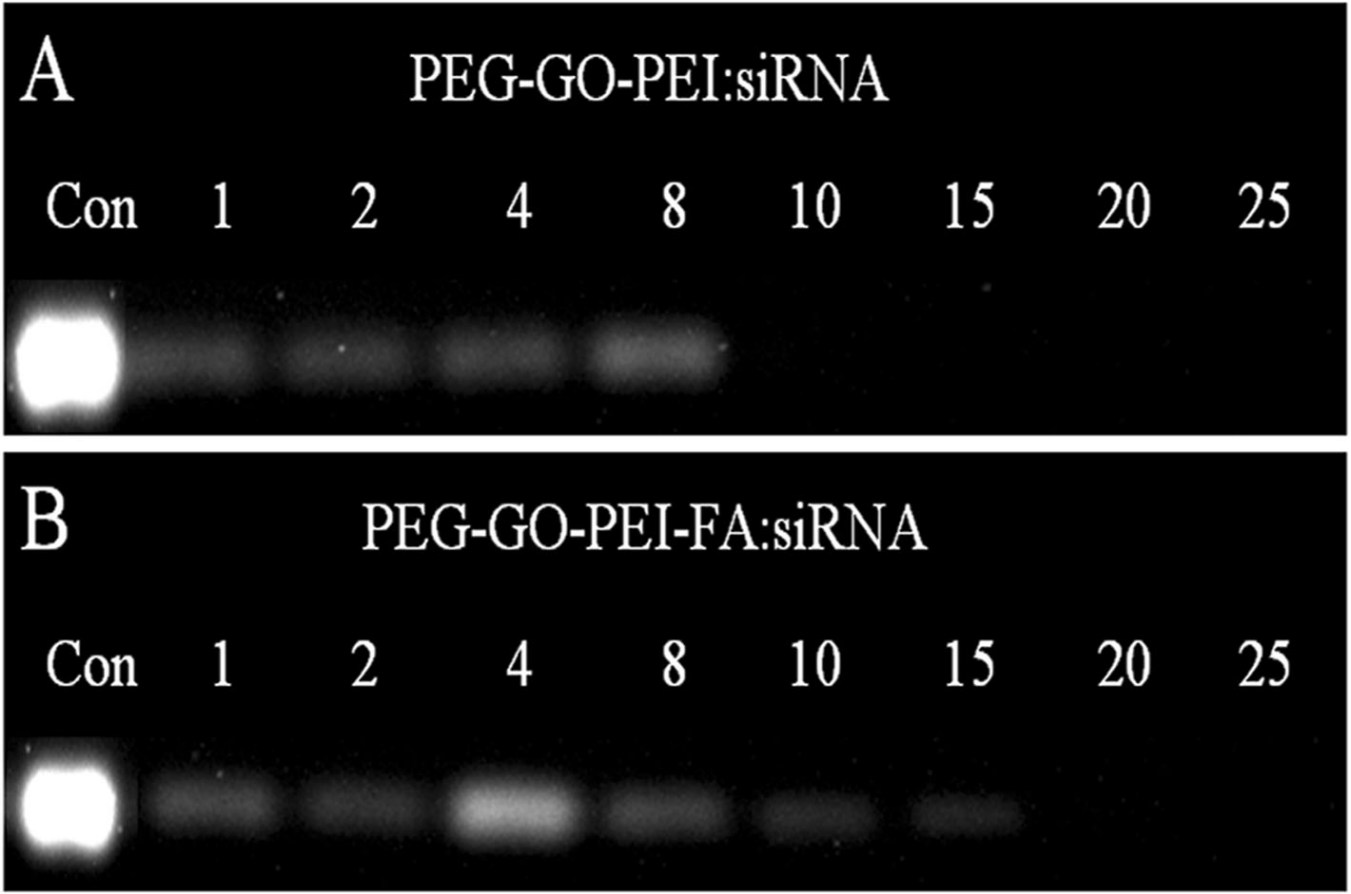
The siRNA-loading capability at different weight ratios. Agarose gel retardation assay was conducted to evaluate the interaction between siRNA and nanocarrier. **a** PEG-GO-PEI and **b** PEG-GO-PEI-FA at the various weight ratios (materials/siRNA)

Cellular uptake of free RNA molecules is usually tricky due to the substantial negative charges that they are bearing [38]. Loading of the negatively charged biomolecules by cationic polymers is widely adopted to solve the problem [39]. In this paper, loading of siRNA on the PEG-GO-PEI-FA vector was achieved by mixing siRNA and PEG-GO-PEI-FA in aqueous solution. As shown in Table 1, the zeta potential of PEG-GO-PEI-FA was + 14.7 mV, indicating that siRNA can be adsorbed to the surface of PEG-GO-PEI-FA by electrostatic interaction. In this study, the gene condensation ability of the nanocomplexes was assessed by agarose gel electrophoresis [40]. Figure 3a showed that PEG-GO-PEI had evident condensation ability towards siRNA at a weight ratio of 10, while the PEG-GO-PEI-FA complete retardation of siRNA migration was observed when the weight ratio reached 20 (Fig. 3b). So, PEG-GO-PEI-FA could protect siRNA from degradation but, at the same time, need a little more nanocomplex to demonstrate good binding ability. This maybe associated with the zeta potentials of PEG-GO-PEI-FA which were not so high. These results indicated that PEG-GO-PEI and PEG-GO-PEI-FA had sufficient delivery ability, especially PEG-GO-PEI-FA, which further exhibited its potential as a valid vector for the efficient and safe delivery of genes.

### Cellular Uptake Analysis

**Fig. 4:**
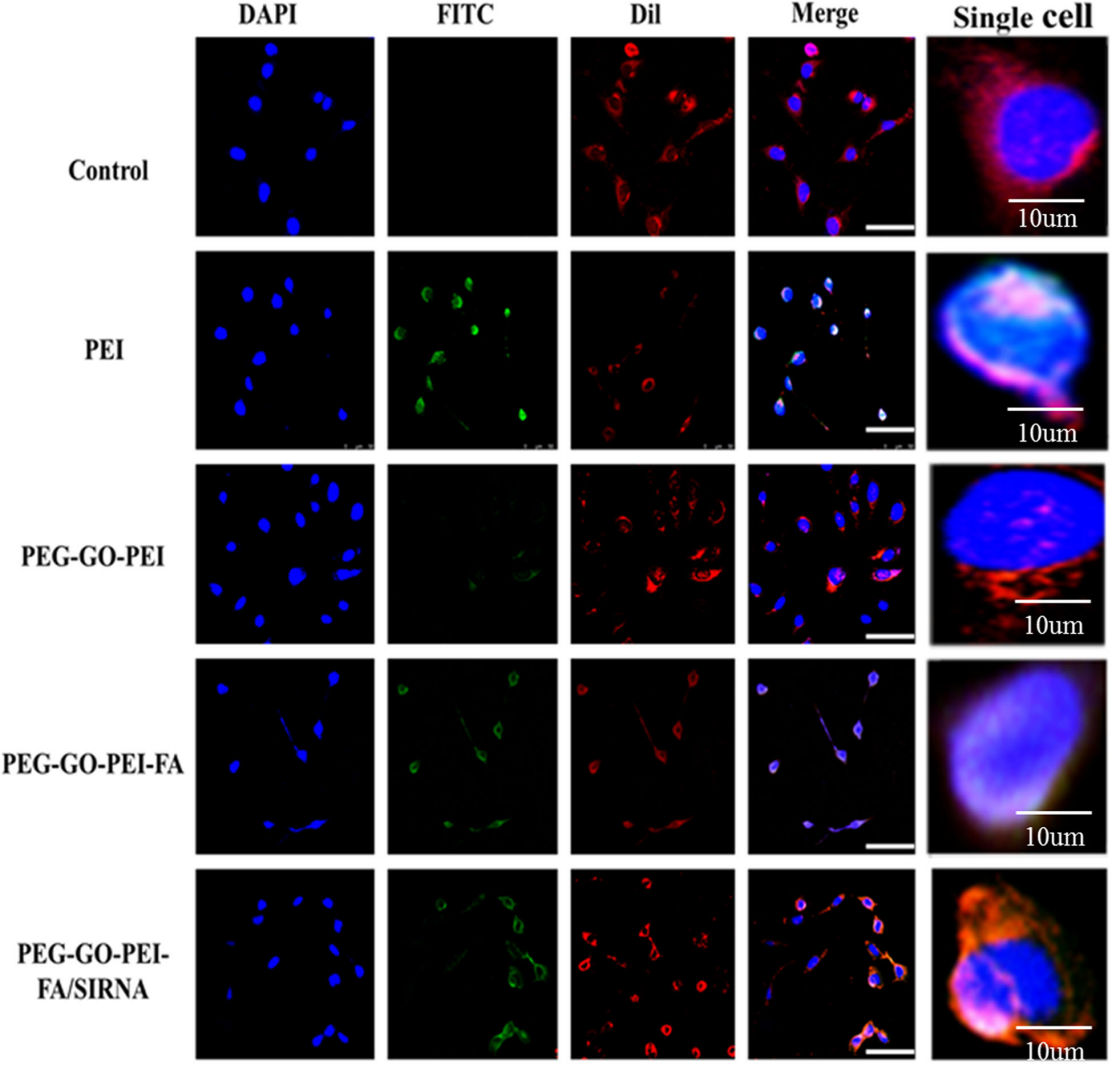
Cellular uptake of different nanocomplexes. Confocal laser scanning microscopy (CLSM) images of the cellular uptake of FITC-labeled different nanocomplexes in human ovarian cancer SKOV3 cells after incubation for 4 h. Blue, nuclei (DAPI-labeled); red, cytomembrane (Dil-labeled); green, nanocomplexes (FITC-labeled). Scale bars represent 100 μm, but the scale bar is 10 μm in single cell

It is so crucial if the carriers can specifically target tumors for gene delivery. In order to confirm whether PEG-GO-PEI-FA carrier could readily enter cells, we used FITC as a fluorescent probe for intracellular imaging. At the same time, the nuclei were stained with DAPI, and the cytomembrane was stained with Dil. As shown in Fig. 4, the control group and PEG-GO-PEI group did not show green fluorescence signals around the nuclei and the cytomembrane. It implied that PEG-GO-PEI did not enter the cells. The PEI 25K and PEG-GO-PEI-FA/siRNA appeared as green fluorescence signal around the core, which clearly demonstrated that PEG-GO-PEI-FA could penetrate cell membranes and enter cells. The PEG-GO-PEI-FA nanocomplexes showed the most active cellular uptake, maybe attributing to the FA can specifically bind FR that overexpressed in the surface of SKOV3 cells. These results suggested that FA played a critical role in mediating the efficient cellular uptake of PEG-GO-PEI-FA and PEG-GO-PEI-FA/siRNA nanocomplexes. More importantly, the ability of FA to bind its receptor was not affected by the covalent amide bond, and the receptor-mediated endocytosis was unhindered.

### Lysosomal Escape Analysis

**Fig. 5:**
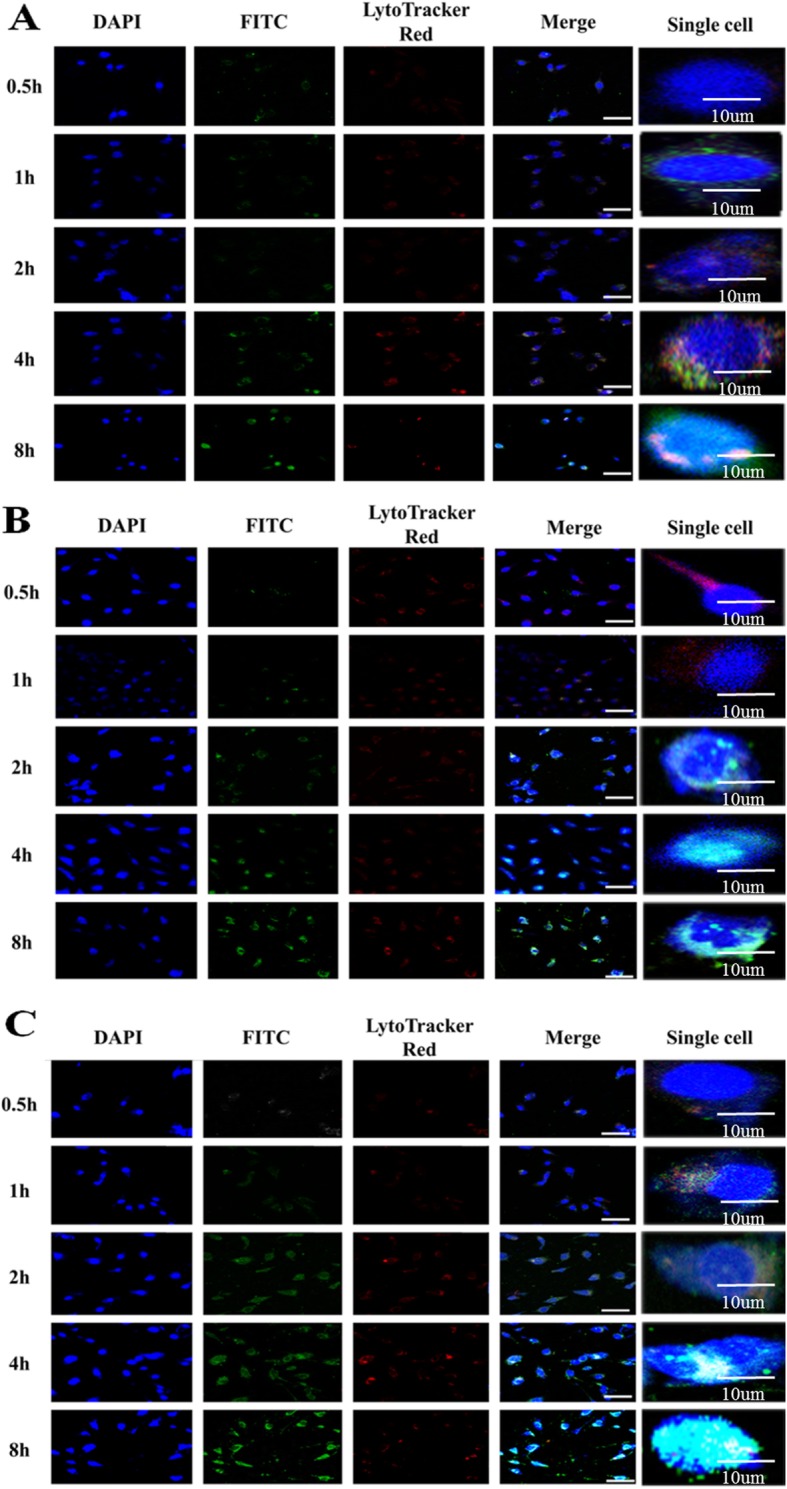
Cellular internalization and lysosomal escape of PEG-GO-PEI-FA observed by CLSM. SKOV3 ovarian cancer cells incubated with **a** PEI, **b** PEG-GO-PEI-FA, and **c** PEG-GO-PEI-FA for 0.5, 1, 2, 4, and 8 h. Those in blue were nuclei stained with DAPI, green were nanocomplexes labeled with FITC, and those in red represented endosomes and lysosome fluorescence after staining with LysoTracker red. Scale bars represent 100 μm, but the scale bar is 10 μm in single cell

For the siRNA delivery system, they should escape from the endosome or formed lysosome. However, if not, the siRNA will degrade or drain out of the cell [41–43]. We evaluate the endosomal escape ability by intracellular localization of the nanocomplexes using CLSM. Since many reports have demonstrated that PEI 25K can favor endosomal or lysosomal escape by “proton sponge effect” [44], so the PEI 25K was used as control. In this study, the nanocomplexes were labeled with FITC. Firstly, we estimated the nanocomplexes enter into the lysosomal by the yellow signal that the green fluorescent signal of FITC overlaying with the red fluorescent signal of Lyto Tracker Red. The PEI 25K appeared as the yellow signal after incubation for 4 h with cells and other materials appeared as the yellow signal after incubation for 2 h with cells, implying the materials had entered into the cells (Fig. 5). Whether or not the nanocomplexes escape from lysosomal by the bright cyan signal, the green fluorescent signal of FITC combines with the blue fluorescent signal of DAPI. As shown in Fig. 5a, the green signal was observed in the accumulation in the nuclei, and there was a bright cyan signal after PEI/siRNA incubation for 8 h with cells, indicating that PEI/siRNA had escaped from the lysosome. However, PEG-GO-PEI-FA and PEG-GO-PEI-FA/siRNA had some weak green signal to penetrate the nuclei as early as 2 h, and greener signal accumulated in the nuclei along with the increase of incubation time. And there was a bright cyan signal when the PEG-GO-PEI-FA and PEG-GO-PEI-FA/ siRNA were incubated for 4 h with cells (Fig. 5b, c). These indicated that the materials had escaped from the lysosome so early. In a word, these results showed that PEG-GO-PEI-FA and PEG-GO-PEI-FA/siRNA remained an excellent endosomal or lysosomal escape ability and can efficiently facilitate lysosomal escape and gene transfection in vitro.

### Cell Inhibitory Evaluation of the PEG-GO-PEI-FA/siRNA

**Fig. 6:**
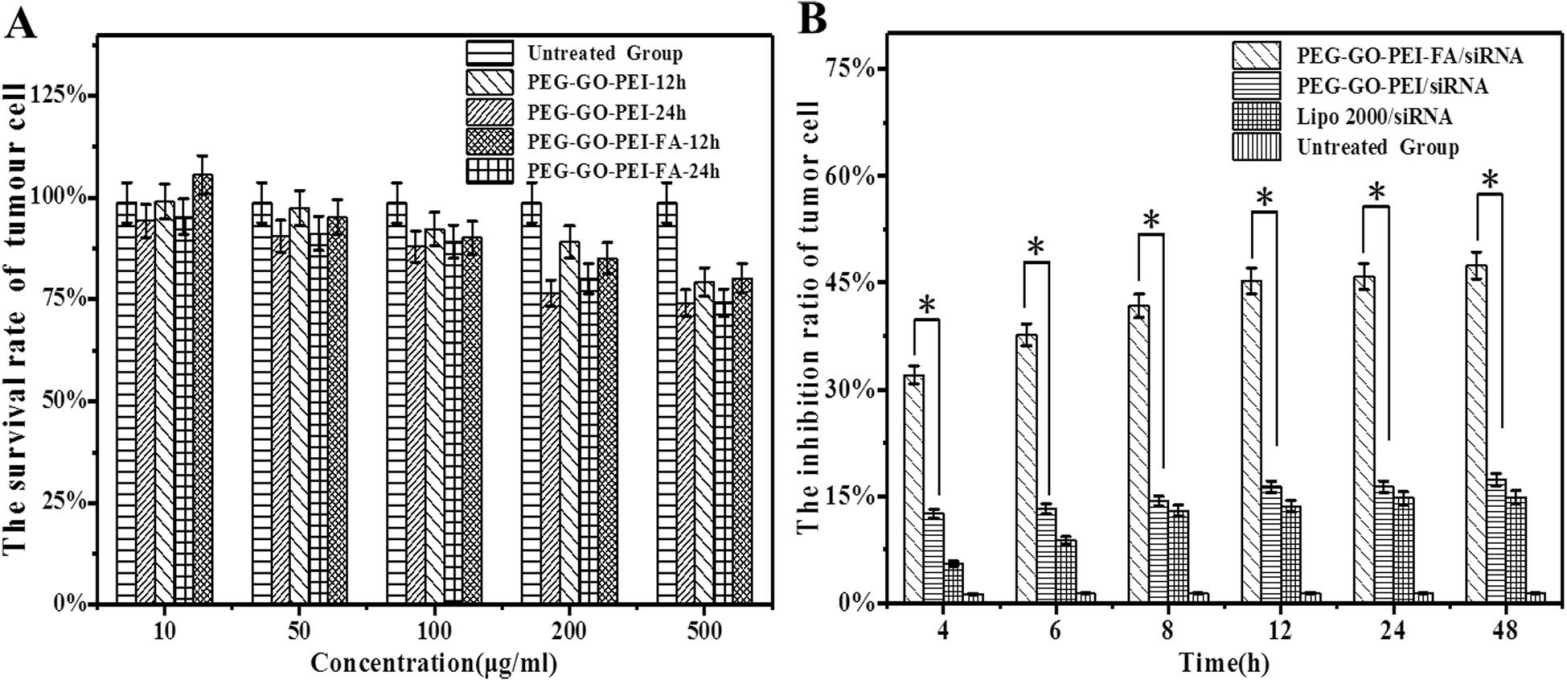
In vitro cytotoxicity of PEG-GO-PEI-FA/siRNA in ovarian cancer SKOV3 cells via CCK-8 assay. **a** SKOV3 cells were treated with PEG-GO-PEI and PEG-GO-PEI-FA at different concentrations (10–500 μg/mL) at 12 and 24 h to get the optimal dose of nanocarrier. **b** The cytotoxicity of PEG-GO-PEI-FA/siRNA, PEG-GO-PEI/siRNA, and Lipo2000/siRNA were measured in different time points (4–48 h) at 100 μg/mL. Error bars represent ± SD; *p < 0.05 (Student’s *t* test)

In the current case, the therapeutic effect of PEG-GO-PEI-FA/siRNA was examined by CCK-8 assay on SKOV3 cells in vitro. As shown in Fig. 6a, we did not find a significant influence on the survival rate of tumor cells at different concentrations (10–100 μg/mL) and different time points (12 and 24 h) in the PEG-GO-PEI-FA group. Even though the concentration was over 100 μg/mL, the survival rate of tumor cells was still more than 80%. So, we chose 100 μg/mL for the next cellular inhibitory study. Weaker cytotoxicity was exhibited in the PEG-GO-PEI/siRNA and Lipo2000/siRNA group (the inhibition rate of less than 20%). Compared with PEG-GO-PEI/siRNA and Lipo2000/siRNA, PEG-GO-PEI-FA/siRNA had a significant inhibitory effect on the growth of SKOV3 tumor cells. PEG-GO-PEI-FA/siRNA inhibited SKOV3 cells in a time-dependent manner (Fig. 6b). These results indicated that PEG-GO-PEI-FA/siRNA had the best inhibition effect for the increment of tumor cells and we could use PEG-GO-PEI-FA as an ideal nanocarrier for gene delivery.

## Conclusions

In this study, we successfully synthesized a novel gene delivery system, PEG-GO-PEI-FA. The not cytotoxic by itself and excellent biological compatibility of PEG-GO-PEI-FA guaranteed its prospects as a safe and effective gene delivery vector. PEG-GO-PEI-FA/siRNA nanocomplexes exhibited outstanding physicochemical properties for gene targeting delivery. Moreover, PEG-GO-PEI-FA/siRNA could readily enter SKOV3 ovarian cancer cells and escape from the lysosomes. Cytotoxicity assay demonstrated that PEG-GO-PEI-FA/siRNA had a good inhibition effect on ovarian cancer cells in a time-dependent manner, and it exhibited a higher cytotoxicity effect compared to other groups. On the basis of aforementioned results, PEG-GO-PEI-FA may provide good anticipation as a gene vector for targeted gene delivery and more effective strategy in ovarian carcinoma treatments.

## Supplementary information

**Additional file 1.** Figure S1. The folate receptor expression in ovarian cancer cells and tissues. (A) The folate receptor expression in different cancer cell lines from Cancer Cell Line Encyclopaedia. (B) The folate receptor expression in tumor and normal ovary tissues (N= 514 samples) from GEPIA2 database and red * indicates p<0.01(the statistic analysis comes from the database).

**Additional file 2.** Figure S2. Schematic illustration. (A) The preparation of PEG-GO-PEI-FA nanoscale delivery system. (B) The therapeutic process of the PEG-GO-PEI-FA/siRNA nanocomplexes in cancer cell.

**Additional file 3.** Figure S3. In vitro biosecurity evaluation of nanocarriers. (A) The cell viability of SKOV3 cells at 24 h after treatment with different concentrations of GO, GO-PEG, PEG-GO-PEI and PEG-GO-PEI-FA. (B) The cell viability of SKOV3 cells at 100 μg/mL after treatment with different time points of GO, GO-PEG, PEG-GO-PEI and PEG-GO-PEI-FA.

## Data Availability

All data generated or analyzed during this study are included in this published article and its supplementary information files.

## Abbreviations

AFM: Atomic force microscope
CCK-8: Cell counting kit8
CCLE: Cancer cell line encyclopedia
CLSM: Confocal laser scanning microscope
DAPI: 4′-6-Diamidino-2-phenylindole
DLS: Dynamic light scattering
DMSO: Dimethyl sulfoxide
DPBS: Dulbecco’s phosphate-buffered saline
EB: Ethidium bromide
EDC∙HCL: 1-(3-Dimethylaminopropyl)-3-ethyl carbodiimide hydrochloride
FA: Folic acid
FBS: Fetal bovine serum
FITC: Fluorescein isothiocyanate
FITR: Fourier transform infrared spectroscopy
FR: Folate receptor
GEPIA: Gene expression profiling interactive analysis
GO: Graphene oxide
MWCO: Molecular weight cutoff
NGO: Nanoscale graphene oxide
NHS: *N*-hydroxysuccinimide
NPs: Nanoparticles
PBS: Phosphate-buffered solution
PDI: Polydispersity index
PEG: Polyethylene glycol
PEI: Polyethyleneimine
siRNA: Small interfering RNA
