# Supplementary figures and images for: Functionalized Folate-Modified Graphene Oxide/PEI siRNA Nanocomplexes for Targeted Ovarian Cancer Gene Therapy
Source: Nanoscale Res Lett. 2020 Mar 6;15:57. doi: 10.1186/s11671-020-3281-7 (PMC7058751; doi:10.1186/s11671-020-3281-7)

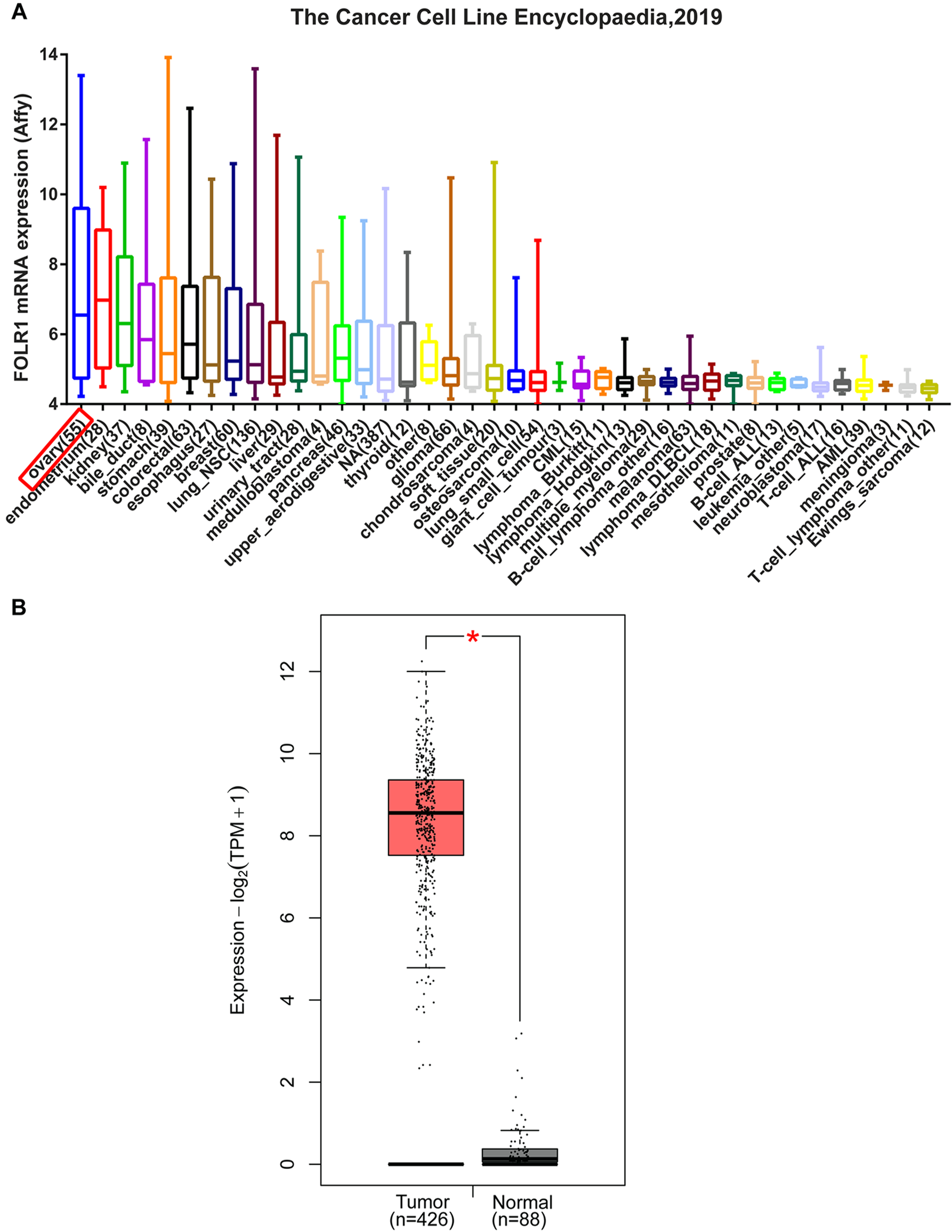

Supplement: Supplementary file 1 — Additional file 1. Figure S1. The folate receptor expression in ovarian cancer cells and tissues. (A) The folate receptor expression in different cancer cell lines from Cancer Cell Line Encyclopaedia. (B) The folate receptor expression in tumor and normal ovary tissues (N= 514 samples) from GEPIA2 database and red * indicates p<0.01(the statistic analysis comes from the database). [file 11671_2020_3281_MOESM1_ESM.tif]

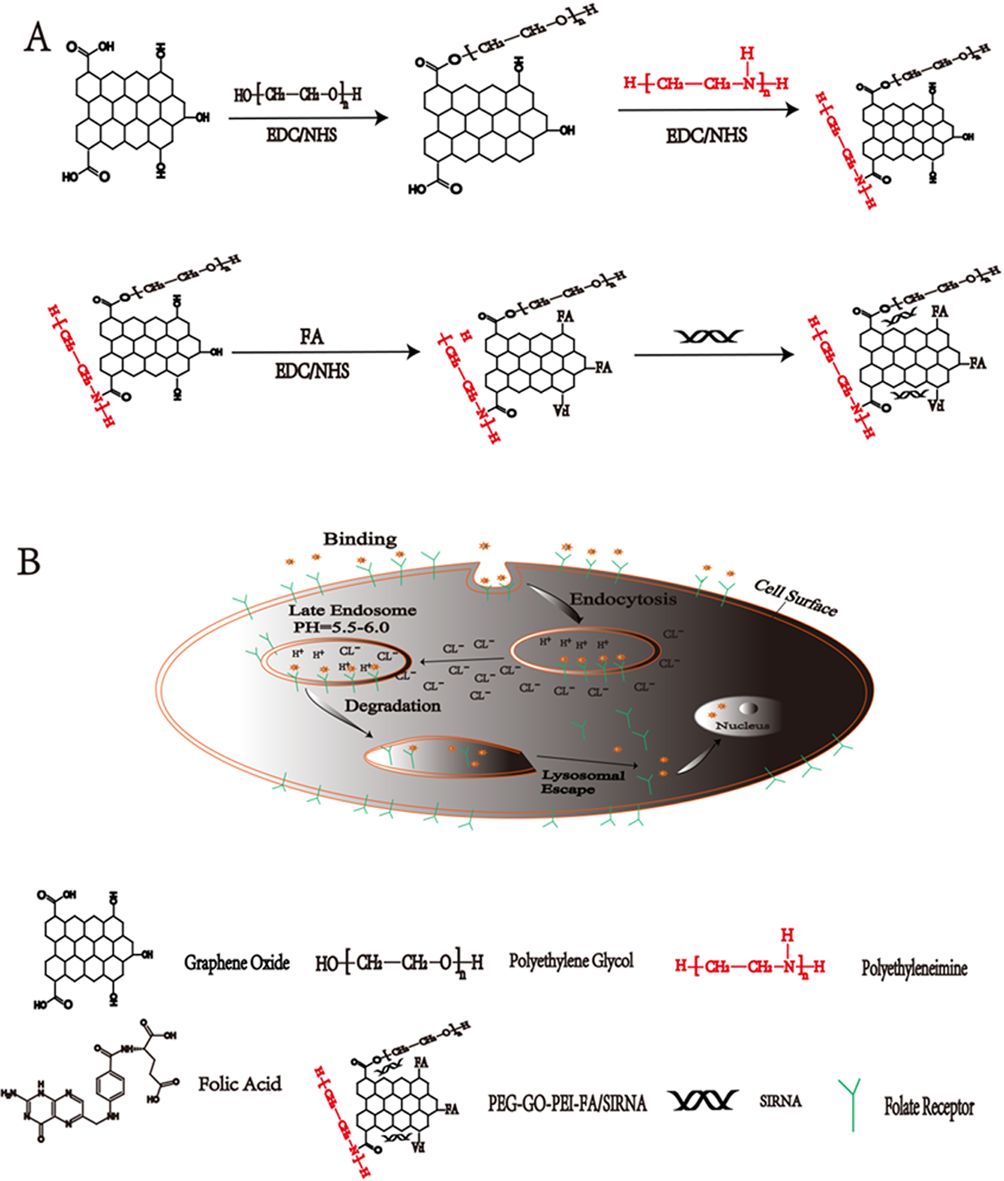

Supplement: Supplementary file 2 — Additional file 2. Figure S2. Schematic illustration. (A) The preparation of PEG-GO-PEI-FA nanoscale delivery system. (B) The therapeutic process of the PEG-GO-PEI-FA/siRNA nanocomplexes in cancer cell. [file 11671_2020_3281_MOESM2_ESM.tif]

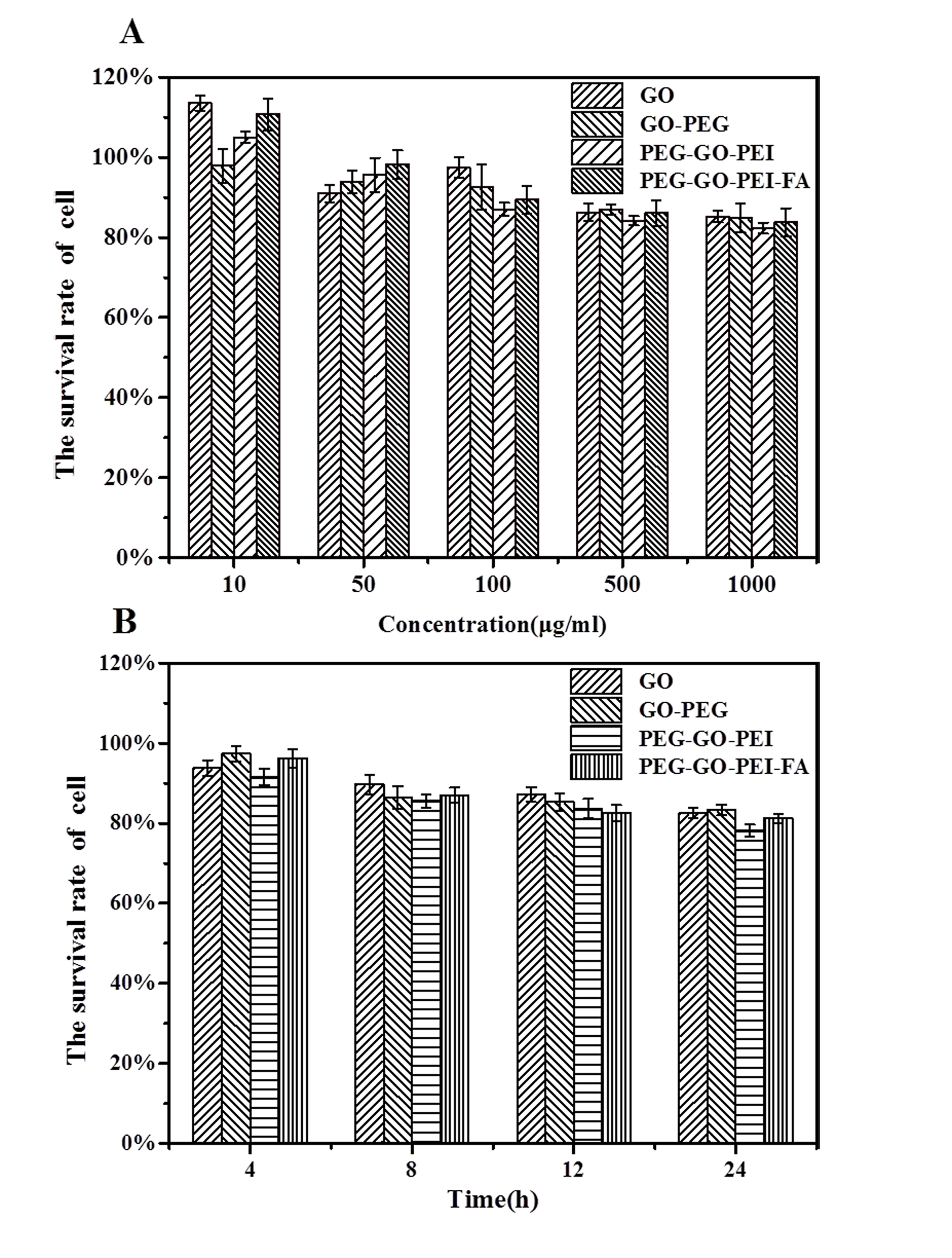

Supplement: Supplementary file 3 — Additional file 3. Figure S3. In vitro biosecurity evaluation of nanocarriers. (A) The cell viability of SKOV3 cells at 24 h after treatment with different concentrations of GO, GO-PEG, PEG-GO-PEI and PEG-GO-PEI-FA. (B) The cell viability of SKOV3 cells at 100 μg/mL after treatment with different time points of GO, GO-PEG, PEG-GO-PEI and PEG-GO-PEI-FA. [file 11671_2020_3281_MOESM3_ESM.tif]
